# Recent research and clinical progress of CTLA-4-based immunotherapy for breast cancer

**DOI:** 10.3389/fonc.2023.1256360

**Published:** 2023-10-04

**Authors:** Hongsheng Zhang, Jintao Mi, Qi Xin, Weiwei Cao, Chunjiao Song, Naidan Zhang, Chengliang Yuan

**Affiliations:** ^1^College of Medical Technology, Chengdu University of Traditional Chinese Medicine, Chengdu, China; ^2^Department of Clinical Laboratory, People’s Hospital of Deyang City, Deyang, China

**Keywords:** CTLA-4, breast cancer, immunotherapy, immune checkpoint inhibitors, clinical trials

## Abstract

Breast cancer is characterized by a high incidence rate and its treatment challenges, particularly in certain subtypes. Consequently, there is an urgent need for the development of novel therapeutic approaches. Immunotherapy utilizing immune checkpoint inhibitors (ICIs) is currently gaining momentum for the treatment of breast cancer. Substantial progress has been made in clinical studies employing cytotoxic T lymphocyte-associated antigen-4 (CTLA-4) inhibitors for breast cancer, but the cure rates are relatively low. To improve the efficacy of CTLA-4-based therapy for breast cancer, further research is imperative to explore more effective immune-based treatment strategies. In addition to monotherapy, CTLA-4 inhibitors are also being investigated in combination with other ICIs or alternative medications. However, it should be noted that immune-based treatments may cause adverse events. This review focuses on the mechanisms of CTLA-4 inhibitor monotherapy or combination therapy in breast cancer. We systematically summarize the latest research and clinical advances in CTLA-4-based immunotherapy for breast cancer, providing new perspectives on the treatment of breast cancer. In addition, this review highlights the immune-related adverse events (irAEs) associated with CTLA-4 inhibitors, providing insights into the development of appropriate clinical tumor immunotherapy regimens and intervention strategies.

## 1 Introduction

According to the International Agency for Research on Cancer’s data from 2020, there were approximately 19.3 million cases of cancer worldwide, resulting in approximately 10 million fatalities (1). The American Cancer Society and the National Center for Health Statistics project that there will be approximately 2 million new cases of cancer and 600,000 fatalities caused by cancer in 2023 (2). Breast cancer incidence rates have shown an upward trend, contrasting with the declining cancer mortality rates since 1991. The reasons for the increasing number of breast cancer cases include poor dietary habits, unhealthy lifestyles and advances in medical technology. Long-term poor dietary habits lead to a decline in physical fitness and affect the hormones levels in the body, thus increasing the incidence of breast cancer. A long-term unhealthy lifestyle leads to endocrine imbalance in the body, increasing the incidence of breast cancer. Advances in clinical medical technology have improved the early detection rate of breast cancer, which may lead to an increase in the reported number of breast cancer cases. Today, breast cancer have surpassed lung cancer as the most frequently malignant tumor and ranks in the top five cancers in terms of mortality (1). The classification of breast cancers is based on the expression levels of the following specific markers: estrogen receptor (ER), progesterone receptor (PR), human epidermal growth factor receptor-2 (HER2), and the Ki-67 index. These classifications include luminal A (ER and/or PR+, HER2-, Ki-67 <20%); luminal B (HER2-negative type/B1: ER+ and/or PR <20%, HER2-, Ki-67 ≥20%; HER2-positive type/B2: ER+ and/or PR+, HER2 overexpression); HER2-positive type (ER- and PR-, HER2+); triple-negative type (ER- and PR-, HER2-); and other specific types of breast cancer (3). These classifications have clear implications for systemic therapy. Adjuvant endocrine therapy is necessary for almost all ER+ breast cancers, while most triple-negative breast cancers (TNBCs) require adjuvant chemotherapy. Moreover, most HER2+ breast cancers require a combination of anti-HER2 therapy and chemotherapy (4). **Table 1** shows the classification of breast cancer.

**Table 1:** Breast cancer classification.

Breast Cancer Type	Molecular Characterization	Treatment	Proportion
Luminal A	ER and/or PR+ HER2- Ki-67 < 20%	Endocrine Therapy	60-70%
Luminal B	(HER2-negative) ER+ and/or PR < 20% HER2- Ki-67 ≥ 20%	Endocrine Therapy	
	(HER2-positive) ER+ and/or PR+ HER2 overexpression	Endocrine Therapy Anti-HER2 Therapy Chemotherapy	
HER2-positive	ER- and PR- HER2+	Anti-HER2 Therapy Chemotherapy	15-20%
TNBC	ER- and PR- HER2-	Chemotherapy	10-17%

Tumors can activate negative regulatory pathways associated with immune homeostasis to effectively suppress immune responses. In recent years, tumor immunotherapy has become a popular research topic. The mechanisms of tumor immunotherapy substantially differ from those of other tumor treatment methods. In contrast to oncogene-targeted therapies, tumor immunotherapy relies on the antitumor response of the tumor-immune cycle and is not limited to targeting individual cancer-promoting factors (5). Among the different types of tumor immunotherapies, immune checkpoint inhibitor (ICI) therapies have the widest impact. In the past, ICIs have been designed to mainly target T cells, but in recent years, natural killer (NK) cells have emerged as targets.

Currently, several antibodies against the cytotoxic T lymphocyte-associated antigen-4 (CTLA-4) or programmed cell death 1 (PD-1)/programmed cell death 1 ligand 1 (PD-L1) signaling pathway are approved for the treatment of various cancers (6). ICIs have achieved significant clinical results in melanoma and non-small cell lung cancer. In recent years, ICIs have also made great progress in treating breast cancer, but there is no systematic summary of the application of CTLA-4 inhibitors in breast cancer. This article focuses on the mechanism of CTLA-4 and the recent research progress of CTLA-4 inhibitors in monotherapy or combination therapy in breast cancer. In addition, we highlight immune-related adverse events (irAEs) associated with CTLA-4 inhibitors. All of these findings provide ideas for the clinical development of appropriate tumor immunotherapy regimens and intervention strategies.

## 2 Immunotherapy and immune checkpoints

An immune response is initiated by immune cells or immune molecules recognizing antigenic foreign bodies and malignant cells, resulting in the elimination of the foreign stimuli and stability of the internal environment (7). However, malignant cells can evade immune surveillance in many ways, proliferate rapidly and then form tumors (8). To change the state of low immunity of the human body, tumor immunotherapy stimulates immune cells to kill tumor cells by artificially enhancing the immune function of the body. Traditional treatments such as chemotherapy or radiotherapy target tumor cells, but the target of immunotherapy is the immune system (9). Specific immunotherapies include vaccination, antibody-specific directed therapy and infusion of specific immune response products. Nonspecific immunotherapies include immune boosters and vaccine suppressants. ICI therapies enhance the antitumor immune response by suppressing immune checkpoints and promoting the activation of T cells, thereby altering the clinical outcome of cancer patients (9). In 2011, ICIs were first applied to melanoma and achieved promising results. Clinical trials have shown that this treatment provides long-term clinical benefits for many cancer patients. In addition to popular immune checkpoints such as CTLA-4 and PD-1/PD-L1, antibodies against other novel immune checkpoints are in clinical development, such as T-cell immunoreceptor with Ig and ITIM domains (TIGIT), T-cell immunoglobulin mucin family member-3 (TIM3) and V-set immunoregulatory receptor (VISTA) (10).

**Figure 1:**
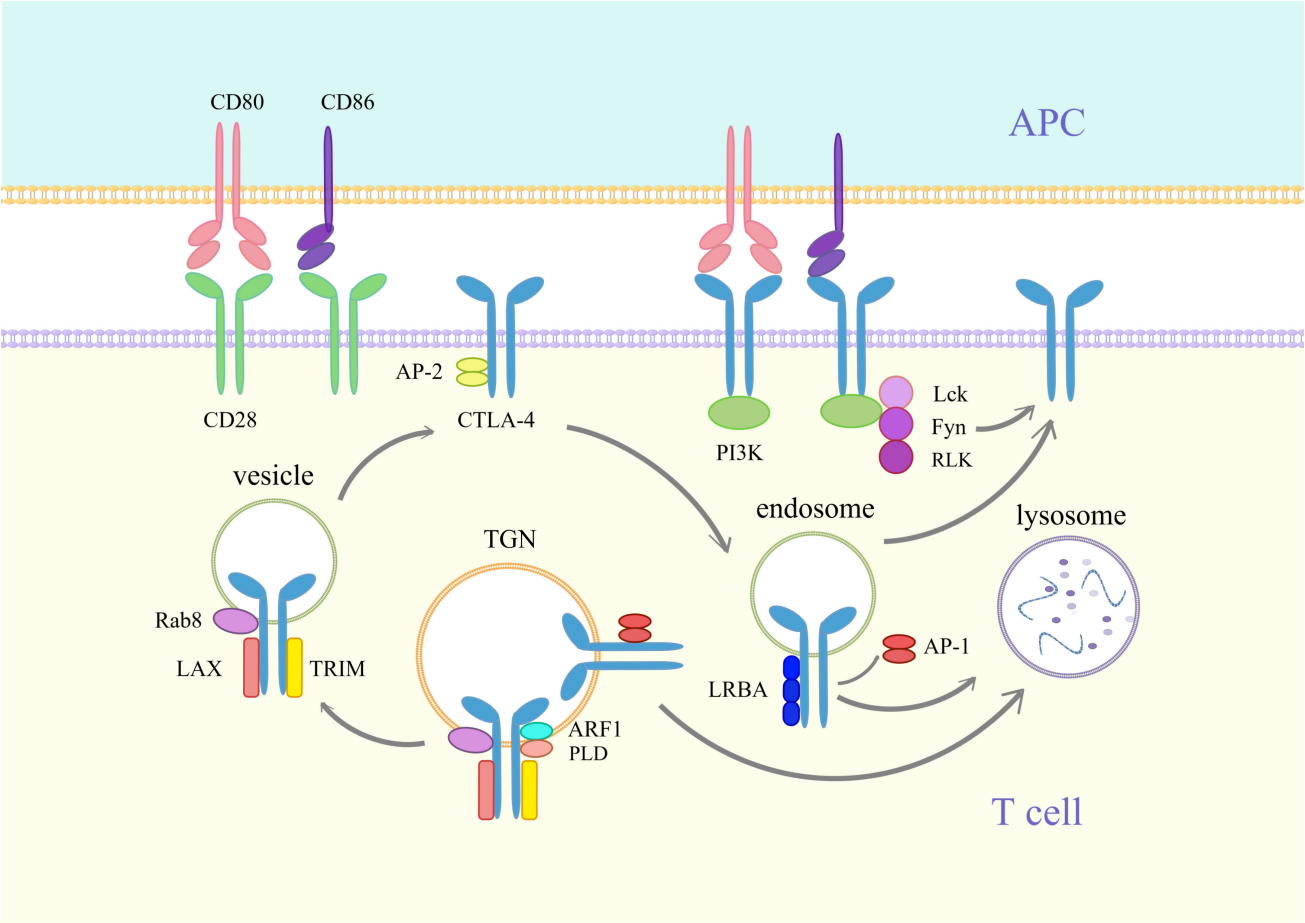
Synthesis and transport of CTLA-4. CD80 is a dimeric ligand, and CD86 is a monomeric ligand. CTLA-4 has a stronger affinity than CD28 and inhibits CD28 binding to CD80/86, thereby blocking T-cell activation. CTLA-4 differs from PD-1 in that it is primarily localized in cytoplasmic vesicles inside the cell. When T cells are activated, CD28 and T-cell receptor (TCR) signaling induce the expression of CTLA-4. Newly synthesized CTLA-4 is transported from the trans-Golgi network (TGN) to the vesicle and cell surface. This process requires the assistance of T-cell receptor interaction molecules (TRIM), the membrane-associated junction protein LAX, the RAS oncogene family Rab8 protein, phospholipase D (PLD), and ADP-Ribosylation Factor 1 (ARF1). The YVKM motif of the surface CTLA-4 cytoplasmic domain can bind to the lattice-protein-associated bridging complex AP-2 and then be internalized into the endosome. Lck, Fyn and RLK kinases can phosphorylate the YVKM motif and inhibit YVKM motif binding to AP-2, thus retaining CTLA-4 on the membrane surface. CTLA-4 in endosomes can bind to Lipopolysaccharide-responsive and beige-like anchor protein (LRBA) and is similarly translocated to the membrane surface. In addition, CTLA-4 in endosomes and TGNs is able to bind to AP-1, resulting in the translocation of CTLA-4 to lysosomes, where it is then degraded.

CTLA-4, also known as CD152, is a transmembrane protein primarily expressed on regulatory T cells (Tregs), CD4+ T cells, and CD8+ T cells. It possesses two cytoplasmic domains with tyrosine-based motifs that are involved in signal transduction. The extracellular surface receptor of CTLA-4 has a structure similar to that of the costimulatory molecule receptor CD28 on the surface of T cells. This structure competitively binds B7-1/2 (CD80/86) molecules on the surface of antigen-presenting cells (APCs) and thus regulates T-cell function and limits excessive immune cell damage (11). CTLA-4 is located within cytoplasmic vesicles and is transported to the cell membrane when the T-cell receptor is activated, facilitated by T-cell interaction molecules (TRIM). Following transport, it undergoes phosphorylation and remains bound to the cell surface in its phosphorylated state. CTLA-4 competes with its counterpart CD28 for binding to CD80 and CD86 ligands, thereby inhibiting the role of CD28 as a costimulatory molecule for T-cell activation (12) (**Figure 1**). Furthermore, due to regulation of reverse endocytosis by CTLA-4, CD80 and CD86 are endocytosed by CTLA-4 from the cell surface into the intracellular compartment, resulting in a decrease in the number of ligands for CD28 (13). In addition to its presence on Tregs and T cells, CTLA-4 has also been confirmed to be expressed in the cytoplasm and on the surface of breast cancer cells (14). The level of CTLA-4 in the serum of breast cancer patients is significantly higher than that in the serum of healthy individuals (15). It has been demonstrated that when CTLA-4+ breast cancer cells and human dendritic cells (DCs) are cocultured, the extracellular signal-regulated kinase and activating transcription Factor 3 (ATF3) of DCs are inhibited, thus suppressing the function and maturation of DCs (16).

PD-1, a transmembrane protein of type 1, is present in various cells, including T cells, B cells, NK cells, and marrow cells. The ligands for PD-1 are PD-L1 and PD-L2, with PD-L1 being the predominant ligand. In the case of T cells bound to antigen, PD-L1 binds to PD-1, which transmits a coinhibitory signal (17). This transmission occurs through tyrosine phosphorylation of PD-1. Consequently, it hinders the activation of T-cell signaling pathways such as PI3K-AKT-mTOR and RAS-MEK-ERK, which impedes T-cell activation and inhibits effector function (18). Moreover, PD-1 has demonstrated its ability to inhibit the cytotoxicity of NK cells (19). PD-L1 is found in several cancer types, including melanoma, colon adenocarcinoma and breast cancer. Notably, higher PD-L1 expression levels are found in TNBC than in luminal and HER2+ breast cancer (20).

**Figure 2:**
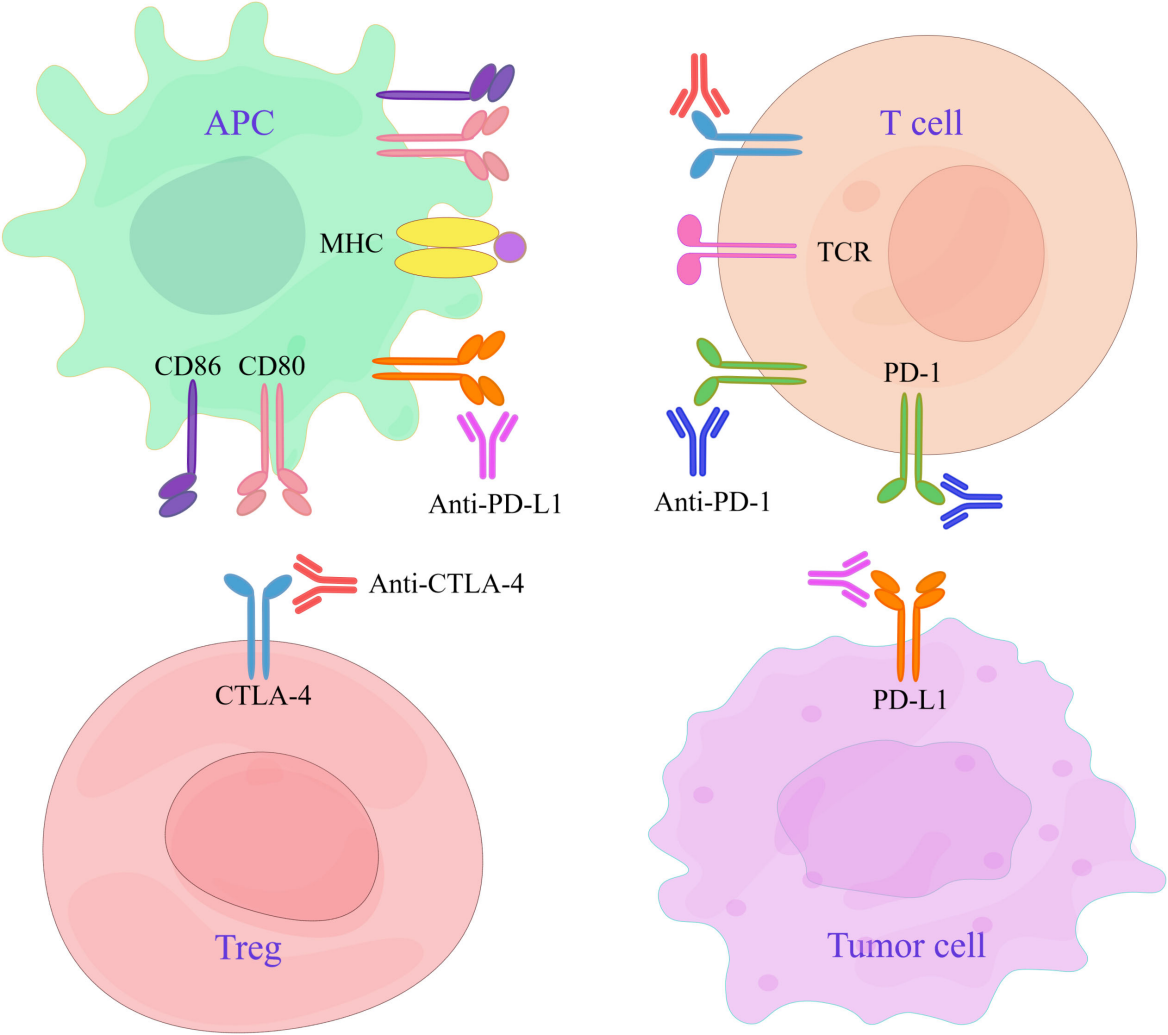
Targets of CTLA-4 and PD-1/PD-L1 inhibitors. APCs interact with Tregs and T cells expressing CTLA-4, and their antigen-presenting ability is reduced. Tumor cells overexpress PD-L1 through various mechanisms, leading to T-cell depletion. CTLA-4 inhibitors can treat cancer by inhibiting CTLA-4 molecules and activating T cells. Anti-PD-1/PD-L1 antibodies are used to restore the killing ability of T cells by blocking the negative signaling pathway and can thus be used to treat cancer.

CTLA-4 and PD-1/PD-L1 are the primary immune checkpoints that exhibit distinct mechanisms of action. The primary function of CTLA-4 is to hinder the interaction between CD28 and CD80/86, thereby regulating the T-cell responses initiated by APCs. However, PD-1 regulates the effective stage of the T-cell reaction mainly by blocking the activation of the T-cell signaling pathway (**Figure 2**). CTLA-4 inhibitors affect clonal expansion and transport of CD4+ T cells. PD-1/PD-L1 inhibitors impact CD8+ T cells but do not influence T-cell clonal expansion or T-cell transport. In general, both inhibitors are designed to improve T-cell function so that T cells can exert their normal antitumor immune effects (21).

PD-1 inhibitors, such as pembrolizumab and nivolumab, are key therapeutic agents for the treatment of breast cancer. Additionally, PD-L1 inhibitors such as atezolizumab and avelumab play an important role in this regard. Moreover, notable advancements in breast cancer therapy have been achieved through the utilization of dual-specific antibodies targeting transforming growth factor-β (TGFR-β) and PD-L1, exemplified by YM101 and BiTP treatment (22, 23). Next, we comprehensively discuss CTLA-4 inhibitors and their role in breast cancer treatment.

## 3 CTLA-4 inhibitor monotherapy

### 3.1 Rationale of anti-CTLA-4 antibodies

Tregs facilitate tumor growth and metastasis. Anti-CTLA-4 antibodies bind with high affinity to CTLA-4 molecules, depleting Tregs and blocking their function. Anti-CTLA-4 antibodies also prevent endocytosis and increases CD80/CD86 levels in APCs. In addition, anti-CTLA-4 antibodies induce antibody-dependent cytotoxicity (ADCC) in tumor macrophages. Most importantly, they directly kill CTLA-4+ breast cancer cells. All of these mechanisms activate T cells, enhance antitumor immunogenicity, and ultimately lead to the death of breast cancer cells.

### 3.2 Anti-CTLA-4 antibodies *in vitro* and *in vivo* experiments

Azimnasab-Sorkhabi et al. performed *in vitro* experiments to demonstrate the effect of anti-CTLA-4 antibody on the migratory and clonogenic capacity of mouse mammary tumor cells (24). In a study by Pruitt et al., the transfection of anti-CTLA-4 antibody into DCs enhanced the activity of cytotoxic T lymphocytes (CTLs), which contributed to the inhibition of breast cancer cell growth (25). To further investigate this, Chen et al. introduced anti-CTLA-4 antibody to cocultures of CTLA-4+ breast cancer cells and DCs. The results showed that DCs produced more cytokines and had greater antigen-presentation capacity, which induced the apoptosis of CTLA-4+ breast cancer cells (16). Furthermore, Grubczak et al. conducted coculture experiments involving lymphocytes and breast cancer cells to explore the role of anti-CTLA-4 antibody. The results revealed that the incorporation of anti-CTLA-4 antibody enhanced the anticancer activity of lymphocytes and significantly arrested breast cancer cells in the G1/S stage of the cell cycle. The study suggests that inhibition of CTLA-4 may improve the action of lymphocytes on breast cancer cells and reduce the proliferation ability of breast cancer cells (26).

Some studies have shown that vascular normalization can enhance tumor immunotherapy (27). In another study, eosinophils were shown to promote tumor vascular normalization (28). Zheng et al. showed that eosinophil accumulation in breast neoplasms and vascular perfusion was improved after the use of anti-CTLA-4 antibody, and this slowed the growth of breast tumors (29). In addition, Rupp et al. showed that anti-CTLA-4 antibody inhibited tumor growth in a mouse TNBC model (30). McCaw et al. found that breast neoplasms expressing MHC class II molecules promoted local activation of CD4+ T cells. When anti-CTLA-4 antibodies were added, tumors expressing MHC II were completely eliminated (31).

### 3.3 CTLA-4 inhibitors

The main CTLA-4 inhibitors used for clinical treatment are ipilimumab and tremelimumab. Ipilimumab, the first ICI approved after being evaluated in metastatic melanoma clinical trials, is a human immunoglobulin IgG1 monoclonal antibody against CTLA-4. Its mechanism involves spatially blocking the interaction between CTLA-4 and CD80/CD86, thereby blocking the suppressive effect of CTLA-4. This blockage allows CD28 to associate with CD80/CD86, leading to T-cell activation (32). Tremelimumab is a human immunoglobulin IgG2 monoclonal antibody against CTLA-4 that shares a comparable mode of action with ipilimumab. It inhibits the binding of CTLA-4 with CD80/CD86, blocks the CTLA-4 pathway, restores the second signal of T cells, increases the number of active effector T cells, and thus enhances the immune attack on tumor cells (33).

### 3.4 Clinical trials evaluating CTLA-4 inhibitors

The safety and efficacy of tremelimumab were assessed in a clinical phase II trial, NCT02527434, involving patients with multiple advanced solid tumors (uroepithelial bladder cancer, pancreatic ductal adenocarcinoma and TNBC). The study found an objective remission rate (ORR) of 8.3% (95% CI: 0.2%-38.5%), median duration of remission (mDOR) of 12.9 months, disease control rate (DCR) of 8.3% at 12 months, median progression-free survival (mPFS) of 3.58 months, and median overall survival (mOS) of 12.88 months in 12 patients with TNBC. Abdominal pain, vomiting, and constipation were the main treatment-related adverse events (trAEs). This study demonstrates that tremelimumab is feasible for the treatment of TNBC. Furthermore, the clinical phase I trial NCT01502592 is currently evaluating the safety of ipilimumab in patients with early-stage mammary gland cancer. An IL-15 antibody that targets the CTLA-4 fusion protein, JK08, is being tested in a phase I/II clinical trial, NCT05620134, in breast cancer patients. In addition to breast cancer, clinical trials of CTLA-4 inhibitors are being conducted in a variety of tumors and include bispecific antibodies against CTLA-4 and PD-1/PD-L1. **Table 2** shows the clinical trials of CTLA-4 inhibitor monotherapy.

**Table 2:** Clinical trials of CTLA-4 inhibitor monotherapy.

Clinical Trials Identifier	Antibody Name	Antibody Type	Indication	Phase	R&D Company
NCT02527434	Tremelimumab	Anti-CTLA-4 Monoclonal Antibody	TNBC and Bladder Cancer	II	AstraZeneca
NCT01502592	Ipilimumab	Anti-CTLA-4 Monoclonal Antibody	Early Stage/Resectable Breast Cancer	I	Memorial Sloan Kettering Cancer Center
NCT05620134	JK08	IL-15 Antibody Fusion Protein Targeting CTLA-4	Breast Cancer and Melanoma	I/II	Salubris Biotherapeutics
NCT04336241	RP2	Encodes Anti-CTLA-4 Antibody Oncolytic Vaccinia Virus	Breast Cancer and Lung Cancer	I	Replimune
NCT03517488	XmAb20717	Anti-PD-1/CTLA-4 Bispecific Antibody	Breast Cancer and Cervical Cancer	I	Xencor
NCT04140526	ONC-392	Humanized Anti-CTLA-4 IgG1 Monoclonal Antibody	Breast Cancer and Esophageal Cancer	I/II	OncoC4
NCT03849469	XmAb22841	Anti-LAG-3/CTLA-4 Bispecific Antibody	TNBC and Pancreatic Cancer	I	Xencor
NCT03369223	BMS-986249	Non-Fucosylated Monoclonal Antibody Against CTLA-4	TNBC and Prostate Cancer	I/II	Bristol-Myers Squibb
NCT04725331	BT-001	Encodes Anti-CTLA-4 Antibody Oncolytic Vaccinia Virus	TNBC and Melanoma	I/II	BioInvent International AB
NCT04501276	ADG116	Anti-CTLA-4 Fully Human Monoclonal Antibody	Advanced/Metastatic Solid Tumors	I	Adagene
NCT03782467	ATOR-1015	Anti-OX40/CTLA-4 Bispecific Antibody	Solid Tumors	I	Alligator Bioscience AB
NCT04645069	ADG126	Full Human IgG1 Antibody Against CTLA-4	Advanced/Metastatic Solid Tumors	I/II	Adagene
NCT04699929	YH001	Anti-CTLA-4 Monoclonal Antibody	Advanced Solid Tumors	I	Eucure (Beijing) Biopharma
NCT03860272	Botensilimab	Fc-Engineered Anti-CTLA-4 Monoclonal Antibody	Angiosarcoma and Melanoma	I	Agenus
NCT04135261	HBM4003	Anti-CTLA-4 VH-Fc Antibody	Melanoma and Hepatocellular Cancer	I	Harbor BioMed US
NCT03761017	Lorigerlimab	Anti-PD-1/CTLA-4 Bispecific Antibody	Prostate Cancer and Colorectal Cancer	I	MacroGenics
NCT04606472	SI-B003	Anti-PD-1/CTLA-4 Bispecific Antibody	Gastric Cancer	I	Sichuan Baili Pharmaceutical
NCT03557918	Tremelimumab	Anti-CTLA-4 Monoclonal Antibody	Urothelial Carcinoma	II	AstraZeneca
NCT04380805	AK104	Anti-PD-1/CTLA-4 Bispecific Antibody	Recurrent/Metastatic Cervical Cancer	II	Akesobio
NCT04469725	KN046	Anti-PD-1/CTLA-4 Bispecific Antibody	Thymic Carcinoma	II	Jiangsu Alphamab Biopharmaceuticals

These clinical trial data were obtained from https://clinicaltrials.gov.

CTLA-4 monotherapy has been successful in cell and animal models of breast cancer and is being evaluated in numerous clinical trials involving patients with a variety of cancers. However, this approach has limitations and benefits for the treatment of patients with specific types of breast cancer. Researchers are combining CTLA-4 inhibitors with other treatments to improve the outcomes of breast cancer patients. For example, anti-CTLA-4 antibodies are used in conjunction with other ICIs, chemotherapy, radiotherapy and local thermotherapy.

## 4 Dual combination therapy with CTLA-4 inhibitors

### 4.1 Dual combination therapy with anti-CTLA-4 and anti-PD-1/PD-L1 antibodies

#### 4.1.1 *In vitro* and *in vivo* trials

Combination therapy shows potential in the medical field, as it has the advantages of both CTLA-4 and PD-1/PD-L1 monotherapy. The mechanism of action for the combination therapy of these two antibodies involves activating the immune system to enhance immune responses and inhibit tumor growth. Anti-CTLA-4 antibodies work by inhibiting CTLA-4 on the surface of T cells, thereby preventing the interaction between CTLA-4 and CD80/86. This disruption promotes the binding of CD28 to CD80/86, ultimately leading to the activation of T cells. Anti-PD-1 antibodies block the interaction between PD-1 on T cells and PD-L1, allowing T cells to more effectively target tumor cells. The synergistic effect of using these two antibodies in combination can be observed. In a study conducted by Rupp et al., a mouse TNBC model was used to evaluate the effects of combining anti-CTLA-4 and anti-PD-1 antibodies. The findings indicated that the combined treatment exhibited the highest efficacy in inhibiting tumor growth, surpassing both the control group and single antibody therapy. Notably, the researchers highlighted the relatively reduced responsiveness of TNBC to immunotherapy compared to other malignancies (34–39). This study suggests that the low sensitivity of TNBC to monotherapy can be overcome by combination therapy (30). A similar investigation by Sun et al. corroborated these results. The combination of anti-CTLA-4 and anti-PD-1 antibodies treatment demonstrated superior outcomes compared to control or single antibody treatment. Specifically, it increased the infiltration of CD4+ and CD8+ T cells within breast tumors, facilitated the accumulation of CD8+ T cells at the tumor margins, decreased tumor size, and reduced incidence of lung metastases. These findings indicate that combination therapy effectively inhibits tumor growth and prevents distant metastasis (40).

#### 4.1.2 Clinical trials

The Life Science Ide Clinical phase II trial NCT02834013 reported the results of ipilimumab in combination with the PD-1 inhibitor nivolumab for the treatment of metaplastic breast cancer (MpBC). MpBC is an aggressive form of breast cancer and does not respond well to chemotherapy and radiation, so the researchers used two ICIs to test their efficacy in MpBC. The results showed that 17 patients with MpBC had an ORR of 18%, mPFS of 2 months, and mOS of 12 months. One patient had complete remission, and two patients had partial remission. Eleven patients (65%) experienced trAEs, most of which were grade 1-2 trAEs, with one grade 5 trAE. This study demonstrated that the combination of these two inhibitors showed promising results in treating MpBC (41). Other studies have shown that the higher the dose of ipilimumab, the more likely trAE is to occur. Therefore, subsequent trials are needed to optimize the dose (42).

Another phase II clinical trial, NCT03789110, also combined ipilimumab and nivolumab in hypermutated HER2- breast cancer. The results showed an ORR of 16.7%, mPFS of 1.4 months (95% CI: 1.3 months-4.6 months), mOS of 19.3 months, and a clinical benefit rate (CBR) of 16.7% in 30 patients. Five patients had grade 5 trAEs. These findings suggest that the combination therapy of ipilimumab and nivolumab is useful but also that dose optimization is needed to improve safety.

Additionally, another phase II clinical trial, NCT02536794, explored the use of tremelimumab in combination with the novel PD-L1 inhibitor MEDI4736 for patients with metastatic HER2- breast cancer. The results revealed that the ORR of the 27 HER2- breast cancer patients was 14.8%. Of the 27 patients, 14 were TNBC patients, and their ORR was 28.6%. The mPFS for the 27 patients was 4.9 months (95% CI: 3.1 months-7.9 months), and the mOS was 11.3 months (95% CI: 7.2 months-36.6 months), with a clinical benefit rate (CBR) of 18.5%. Among all patients, seven experienced grade 3 to 4 trAEs, with no cases of grade 5 trAE. These results indicate that the combination of these two immune inhibitors offers a new therapeutic option for breast cancer.

Currently, a phase II clinical trial, NCT03982173, investigating the combination therapy of tremelimumab and the PD-L1 inhibitor durvalumab for TNBC has been terminated, but another clinical trial is still ongoing. The early-phase I clinical trial, registered as NCT03132467, aims to evaluate the feasibility and tolerability of these two inhibitors in HR+/HER2- breast cancer patients.

### 4.2 Dual combination therapy with anti-CTLA-4 antibodies and other drugs

#### 4.2.1 *In vitro* and *in vivo* trials

Apart from the combined use of two ICIs, anti-CTLA-4 antibodies have also been employed in conjunction with other drugs for the treatment of breast cancer. Cytokines can enhance tumor immunogenicity and promote tumor immunotherapy (43). Qu et al. discovered that IL-36 can stimulate the proliferation of CD4+ and CD8+ T cells, but it can also promote the expansion of Tregs. The results demonstrated an increase in the expression level of IFN-γ and an improvement in breast tumor cell metastasis to the lungs. The combined application of IL-36 with anti-CTLA-4 antibody is beneficial for depleting Tregs and increasing the antitumor activity of IL-36. Thus, this combination therapy can enhance the clinical response to immunotherapy in breast cancer (44). The combined mechanism of action of these two factors involves promoting the activation and proliferation of T cells and NK cells, enhancing the infiltration of immune cells within the TME, inducing the production of various proinflammatory cytokines, and contributing to the establishment of prolonged immune memory.

To enhance the efficacy of chemotherapy, Maulhardt et al. developed submicron particle-loaded docetaxel (SPD). These submicron particles can carry a large amount of docetaxel and exhibit significantly better efficacy than free docetaxel (45). They also described the immunomodulatory characteristics of SPD when combined with anti-CTLA-4 antibody for breast cancer treatment. In the mammary tumors of mice, the combination resulted in a significant increase in the number of NKT cells and CD4+ T cells, an increase in B cell numbers in peripheral blood, and a decrease in the number of suppressor cells derived from the bone marrow. This result indicates that the combination of SPD and anti-CTLA-4 antibody induces antitumor immunogenicity (46). When anti-CTLA-4 antibody isused in combination with chemotherapy, the actions of both treatment approaches synergize. Anti-CTLA-4 antibody enhances the immune response of the immune system, enabling T cells to more effectively attack tumor cells. Chemotherapy, in contrast, reduces tumor volume, lessening the burden on the immune system, and kills tumor cells, potentially triggering a stronger immune response. The goal of this combination therapy is to enhance the overall treatment effect, enabling the patient’s immune system to more effectively combat cancer.

Solid tumors exhibit sensitivity to local hyperthermia. Local hyperthermia works by damaging tumor cell membranes and releasing tumor-specific antigens, thereby triggering the immune system’s response against the tumor. Consequently, local hyperthermia has emerged as a novel adjunctive therapy for tumors (47). Ibuki et al. conducted a study in which mice were inoculated with breast cancer cell lines on both sides of their hind limbs and treated with a combination of anti-CTLA-4 antibody and local hyperthermia. Compared to monotherapy, the combination therapy resulted in increased survival of mice and smaller tumor volumes, indicating that local hyperthermia can enhance the efficacy of anti-CTLA-4 antibody treatment and improve the antitumor response (48). Anti-CTLA-4 antibody alleviates T-cell inhibition, while local hyperthermia induces localized immune stimulation, prompting a stronger immune response against the tumor. These two treatment modalities complement each other, enhancing the potential effects of immune cells and thus more effectively eliminating tumor cells.

Radiation therapy uses high-energy radiation to directly kill or damage tumor cells, resulting in the release of tumor-specific antigens, which can be recognized by the immune system as foreign substances, triggering an immune response. The use of anti-CTLA-4 antibody prevents T cells from being inhibited, enabling them to better identify and attack tumor cells. To improve the effectiveness of radiation therapy, Dewan et al. evaluated the efficacy of single-dose or fractionated radiation therapy in combination with anti-CTLA-4 antibody. These results show that fractionated radiotherapy combined with anti-CTLA-4 antibody had a greater efficacy in treating breast tumors, with increased production of IFN-γ and smaller tumor volumes, surpassing the effectiveness of single-dose radiation therapy in combination with anti-CTLA-4 antibody. This experiment provides a reference for the clinical application of combined radiation therapy and immune therapy (49).

Metformin has been reported to significantly inhibit tumor growth and enhance the immune activity of CTLs (50). It exerts its effects through three primary mechanisms. Metformin activates AMP-activated protein kinase (AMPK), an energy-sensing protein kinase, which can inhibit the intracellular insulin signaling pathway, reducing tumor cell glucose uptake and utilization. Metformin can decrease the number of Tregs, thereby alleviating immune response suppression. Additionally, it can enhance cytokine production, promoting immune cell attacks on tumor cells. The mechanism of action for the combined treatment of anti-CTLA-4 antibody and metformin in tumors involves the alleviation of T-cell inhibition, enhancement of T-cell antitumor immune activity, and regulation of tumor cell metabolism, achieving a synergistic antitumor effect. Cha et al. applied metformin and anti-CTLA-4 antibody to a 4T1 breast tumor model in mice. They observed a significant reduction in tumor size, increased CTL activity, improved mouse survival rates, and no toxicity detected in the kidneys and liver. These results indicate that metformin can enhance the treatment efficacy of anti-CTLA-4 antibody in breast cancer (51).

The novel anticancer drug RX-5902 can block the β-catenin signaling pathway (52). Tentler et al. evaluated the efficacy of combining anti-CTLA-4 antibody with RX-5902 in a TNBC mouse model. This combination led to increased T-cell activation and stronger tumor growth inhibition. The mice showed suitable tolerance without adverse reactions. These findings suggest that blocking the β-catenin signaling pathway can enhance the immune response to anti-CTLA-4 antibody (53).

High levels of folate receptors are expressed in many tumors, leading to the development of a new approach using folate-radioactive drugs to enhance antitumor immunity (54). Guzik et al. applied [177Lu]Lu-DOTA-folate and anti-CTLA-4 antibody to mice with breast cancer. They observed that [177Lu]Lu-DOTA-folate could bind to tumor cells *in vivo*, accumulate in the tumor, and make it sensitive to treatment. When combined with anti-CTLA-4 antibody, tumor growth was suppressed, and the survival time of mice was significantly prolonged. These results indicate that [177Lu]Lu-DOTA-folate promotes the antitumor effects of anti-CTLA-4 antibody (55).

A novel HER2-targeting antibody–drug conjugate, [Fam-] trastuzumab deruxtecan, has demonstrated potent antitumor activity in various tumors (56). It has been reported that [Fam-] trastuzumab deruxtecan in conjunction with anti-PD-1 antibody has been successfully used in a mouse colon cancer model (57). Iwata et al. tested the effects of [Fam-] trastuzumab deruxtecan in combination with anti-CTLA-4 antibody in a mammary tumor mouse model. The results showed extensive infiltration of CD4+ and CD8+ T cells into the tumors, indicating a more potent antitumor immune response induced by the combination therapy (58).

Aside from the previously mentioned therapeutic strategies, there exists an additional group of immunotoxins designed to target CTLA-4, which has gained widespread usage (59). This approach involves combining the targeting specificity of anti-CTLA-4 antibodies with a cytotoxic payload, often composed of bacterial or plant toxins. These toxins specifically eliminate Tregs expressing elevated levels of CTLA-4 on their surface. By neutralizing Tregs, the immunotoxins augment the immune system’s capacity to trigger a potent antitumor reaction (60–62). Both SS1P and LMB-100, immunotoxins directed at mesothelin, have undergone assessment in multiple clinical trials for cancer therapy. In their study, Leshem et al. validated the combined impact of SS1P or LMB-100 alongside anti-CTLA-4 antibody treatment in the 66C14 BALB/c mouse mammary carcinoma cell line. Their findings revealed that this joint administration suppressed CTLA-4 expression levels, leading to complete regression of 86% of the injected tumors and 53% of the noninjected tumors. This regression was accompanied by the initiation of antitumor immunity (63). Not limited to breast cancer, CTLA-4-targeting immunotoxins displayed analogous effects in the AE17-M mesothelioma model (64). Looking ahead, the future of CTLA-4-targeted immunotoxins is promising. Through the selective targeting and elimination of Tregs within the TME, they offer the potential to amplify the effectiveness of ICI therapy. Nonetheless, obstacles persist, including the management of potential off-target effects and the optimization of the equilibrium between enhancing antitumor immunity and preventing excessive immune activation. More research and clinical trials are needed to refine the design and application of these immunotoxins and develop more efficient and focused approaches to cancer treatment.

#### 4.2.2 Clinical trials

A phase I clinical trial, NCT01502592, investigated the tolerability of combined treatment with ipilimumab and cryoablation in patients with breast cancer. The results revealed an elevation in Th1 cytokine levels and T-effector cell numbers in the patient. Among the 19 patients, only one case experienced a grade 3 trAE, indicating the safety of this therapeutic approach, which exhibits robust immune effects (65). **Table 3** demonstrates the clinical trials of dual combination therapy with CTLA-4 inhibitors.

**Table 3:** Clinical trials of dual combination therapy with CTLA-4 inhibitors.

Clinical Trials Identifier	Breast Cancer Type	Phase	Therapeutic Drug	ORR/%	mPFS/ months	mOS/ months	CBR/%
NCT02834013	Metaplastic	II	Ipilimumab Nivolumab	18.0	2.0	12.0	—
NCT03789110	Metastatic Hypermutated HER2-	II	Ipilimumab Nivolumab	16.7	1.4 (95% CI: 1.3-4.6)	19.3	16.7
NCT02536794	Metastatic HER2-	II	Tremelimumab MEDI4736	14.8	4.9 (95% CI: 3.1-7.9)	11.3 (95% CI: 7.2-36.6)	18.5
NCT03982173	TNBC	II	Tremelimumab Durvalumab	—	—	—	—
NCT03132467	HR+/HER2-	I	Tremelimumab Durvalumab	—	—	—	—
NCT01502592	Invasive Adenocarcinoma	I	Ipilimumab Cryoablation	—	—	—	—

These clinical trial data were obtained from https://clinicaltrials.gov.

Dual combination therapy targeting CTLA-4 has been shown to be successful in mice or patients who have failed monotherapy with anti-CTLA-4 antibodies. This finding suggests that combination therapy can overcome adaptive resistance mechanisms and achieve higher response rates. Compared to monotherapy, dual combination therapy optimizes the treatment scope and efficacy. However, there remains substantial room for improvement in increasing response rates.

## 5 Multiple combination therapy with CTLA-4 inhibitors

### 5.1 Multiple combination therapy with anti-CTLA-4 antibodies, anti-PD-1/PD-L1 antibodies, and other drugs

#### 5.1.1 *In vitro* and *in vivo* trials

Researchers have extended their investigations beyond dual therapies with anti-CTLA-4 and anti-PD-1/PD-L1 antibodies, incorporating additional drugs to enhance immune responses. In previous studies, Blomberg et al. observed an increase in the Treg population in mice with spontaneous breast cancer when treated with a combination of anti-CTLA-4 and anti-PD-1 antibodies (66). They further applied a short-term diphtheria toxin (DT) and combined it with anti-CTLA-4 and anti-PD-1 antibodies in a mouse model of breast cancer. DT was able to deplete Tregs. Following the combination therapy, there was a significant increase in the numbers of NK cells and CD8+ T cells in the mice. Even after the discontinuation of the treatment, T cells continued to exhibit sustained activation. These findings demonstrate that combination therapy with anti-CTLA-4 antibody, anti-PD-1 antibody, and DT can inhibit Tregs, activate lymphocytes, and promote immune responses in breast cancer model mice (67).

Due to the ability of innate immune activators to enhance antitumor activity, they have become a popular research topic. Gonzalez et al. investigated the feasibility of combining a TLR5 agonist with anti-CTLA-4 and anti-PD-1 antibodies in a 4T1 mouse model of breast cancer. The mice showed significantly increased survival rates, and upon rechallenge with cancer cells, they exhibited immune memory. These outcomes suggest that innate immune activators contribute to improving the treatment efficacy of ICIs in breast cancer (68).

The tumor microenvironment (TME) undergoes metabolic changes that can lead to an acidic environment, impairing the body’s immune response (69). Chafe et al. found high levels of carbonic anhydrase IX (CAIX) in a mouse model of basal-like breast cancer. They were able to reduce tumor acidification by targeting CAIX using a small molecule called SLC-0111. Furthermore, by combining SLC-0111 with anti-CTLA-4 and anti-PD-1 antibodies, they demonstrated that the triple therapy could target tumor-induced central necrosis and reduce the burden of lung metastasis (70).

Researchers have also combined oncolytic adenoviruses with ICIs. In a 4T1 mouse model of breast cancer, Yang et al. found that triple therapy with anti-CTLA-4 antibody, anti-PD-1 antibody, and an oncolytic adenovirus downregulated the expression of tumor growth-related genes, upregulated perforin and granzyme expression, and suppressed liver and lung metastasis of breast tumors. Similarly, Zhang et al. discovered that this triple therapy increased the number of CD8+ T cells and T memory cells around the tumor, promoted polarization of macrophages from an M2 to M1 phenotype, inhibited breast tumor growth, and prolonged the survival of mice. These two studies indicate that oncolytic adenoviruses serve as enhancers of immune therapy for breast cancer (71, 72).

Antifibrotic drugs can normalize tumor stroma, thereby improving the clinical efficacy of treatment (73, 74). In their study, Panagi et al. investigated whether the low-dose cytotoxic drug Doxil could enhance the effectiveness of the antifibrotic drug tranilast and optimized anti-CTLA-4 and anti-PD-1 antibodies immunotherapy by combining tranilast and Doxil. When immunotherapy was combined with Doxil in a mouse TNBC model, 40% of the tumors showed inhibited growth, while the combination of immunotherapy and tranilast resulted in 50% tumor growth inhibition. The smallest tumor volume was observed after treatment with tranilast + Doxil + ICIs, indicating that concurrent administration of antifibrotic and cytotoxic drugs during immunotherapy can significantly enhance immunogenicity (75).

To further elucidate the response of TNBC to combination immunotherapy and chemotherapy, Napier et al. evaluated the changes in tumor hypoxia and effector cell activation using [18F]-fluoromisonidazole (FMISO) positron emission tomography (PET) imaging and granzyme B-specific positron emission tomography (GZP-PET) imaging. Compared to the control treatment, the combination treatment of paclitaxel (PTX) + anti-CTLA-4 antibody + anti-PD-1 antibody inhibited tumor cell proliferation, improved CD4+ T cell proliferation, reduced hypoxia, with notable improvements in vascular distribution and significant increases in IL-2, IL-4, and IL-12 levels. This finding suggests that FMISO-PET and GZP-PET imaging can better monitor the efficacy of immunotherapy combined with chemotherapy in breast tumors (76, 77).

PI3K is highly expressed in breast cancer. Preventing mammary tumor growth can be achieved by PI3K inhibitors (78). Yan et al. aimed to evaluate the tumor growth response to PI3K inhibitors, PTX, anti-CTLA-4 antibody, and anti-PD-1 antibody treatment. Activation of CD8+ T cells increased in the breast cancer mouse model, leading to significant inhibition of breast cancer cell and tumor growth. CD8+ T cells, dendritic cells, and NK cells were consistently induced to produce responses. The combination of anti-CTLA-4 antibody + anti-PD-1 antibody + chemotherapy + PI3K inhibitors may be a novel treatment for breast cancer (79).

Immunotherapy is often combined with radiotherapy in cancer treatment to enhance antitumor immune responses (80). Grid radiation treatment (GRID) is a radiation technique that delivers different radiation doses to large tumor volumes (81). Johnsrud et al. investigated whether GRID could enhance the immune activity of anti-CTLA-4 antibody + anti-PD-1 antibody therapy in a TNBC mouse model. In mice receiving whole-body irradiation with combined immunotherapy, irradiated tumor growth was inhibited, but distant tumors continued to expand. After receiving spatially segmented irradiation combined with immunotherapy, contralateral tumors exhibited a high presence of APCs and activated T cells, accompanied by upregulated systemic IFN-γ expression and suppressed tumor growth in various body sites. The combination of GRID and ICIs is emerging as a new approach for antitumor therapy (82).

It has become a consensus that exercise can prevent disease occurrence and improve the effectiveness of tumor treatment (83). However, the impact of exercise on immunogenicity remains unclear. Santos et al. studied the changes in immune cells during exercise training (ExTr) in a breast cancer mouse model and examined the alterations in immune response capacity with the combination of anti-CTLA-4 antibody, anti-PD-1 antibody, and ExTr. The addition of anti-CTLA-4 antibody and anti-PD-1 antibody in ExTr significantly increased the populations of granzyme B+ CD8+ T cells and IFNγ+ CD8+ T cells. The triple combination therapy demonstrated excellent ability in controlling breast tumor expansion. These findings demonstrate that the addition of ExTr to immunotherapy can improve the treatment of breast cancer (84).

#### 5.1.2 Clinical trials

A phase I/II clinical trial, NCT02643303, investigated the combination of tremelimumab, the PD-1 inhibitor durvalumab, and the TME modulator Poly-ICLC in breast cancer patients. The results revealed a DCR of 21.4% among 15 patients, with an mPFS of 2.8 months (95% CI: 1.4 months - 7.5 months) and an mOS of 10.8 months. Three patients succumbed to the disease, while the remaining patients experienced mild trAE. These results indicate the feasibility of this triple combination therapy in breast cancer; however, further improvements in safety are necessary. Furthermore, there was a phase II clinical trial, NCT03430466, which added the ER antagonist fulvestun to the immunotherapy of tremelimumab and durvalumab in patients with HR+/HER2- breast cancer, but the trial was terminated for unknown reasons. In addition, an ongoing phase I/II clinical trial, NCT03518606, is currently investigating the efficacy of tremelimumab + durvalumab + the chemotherapeutic agent metronomic oral vinorelbine in the treatment of breast cancer. Another phase II clinical trial, NCT03606967, is evaluating the efficacy of tremelimumab + durvalumab + chemotherapeutic agent nab-paclitaxel ± personalized synthetic long peptide vaccine (neoantigen vaccine) in treating TNBC.

Enzymes involved in cell growth can be disrupted by entinostat and selinexor, leading to tumor cell death. A phase I clinical trial, registered as NCT02453620, is investigating the preliminary antitumor activity of ipilimumab, nivolumab, and entinostat in breast cancer. Similarly, another phase I clinical trial, NCT02419495, is assessing the efficacy of ipilimumab, nivolumab, and selinexor combination therapy in breast cancer patients. Talimogene laherparepvec, an oncolytic virus, can enhance the immunogenicity of the TME. A phase I clinical trial, NCT04185311, is investigating the efficacy of ipilimumab, nivolumab, and talimogene laherparepvec in patients with TNBC or ER+/HER2- localized breast cancer. Trials combining cryoablation with two ICIs are also underway. Clinical trial NCT02833233 is investigating the use of ipilimumab, nivolumab, and cryoablation in the treatment of early-stage breast cancer. Additionally, a phase II clinical trial, registered as NCT03546686, is utilizing ipilimumab, nivolumab, and cryoablation for the treatment of TNBC. Furthermore, an ongoing phase II clinical trial, NCT03409198, is applying the combination of two chemotherapeutic agents, pegylated liposomal doxorubicin and cyclophosphamide, along with ipilimumab and nivolumab in patients with HR+ breast cancer. Glembatumumab vedotin (CDX-011) is an antibody–drug conjugate that delivers the drug into cancer cells via glycoprotein NMB (gpNMB), resulting in cancer cell death. The efficacy of CDX-011 in the treatment of breast cancer was demonstrated in the clinical trial NCT01997333. However, the phase I/II clinical trial NCT03326258 using ipilimumab + nivolumab + CDX-011 for solid tumors such as melanoma and TNBC has been withdrawn.

INT230-6 consists of a combination of two validated anticancer drugs along with a penetration-enhancing molecule. The efficacy of the triple therapy comprising ipilimumab, pembrolizumab, and INT230-6 is being evaluated in a phase I/II clinical trial NCT03058289 among patients with breast cancer. Additionally, a phase II/III clinical trial, NCT03755739, is assessing the safety and differences of ipilimumab, pembrolizumab, and chemotherapy drugs (such as doxorubicin) when administered through arterial or intratumoral infusion in breast cancer patients. **Table 4** shows the clinical trials of multiple combination therapies involving CTLA-4 inhibitors.

**Table 4:** Clinical trials of multiple combination therapies with CTLA-4 inhibitors.

Clinical Trials Identifier	Breast Cancer Type	Phase	Therapeutic Drug	ORR/ %	mPFS/ months	mOS/ months	CBR/ %
NCT02643303	All Types	I/II	Tremelimumab Durvalumab Poly-ICLC	—	2.8	10.8	—
NCT03430466	HR+/HER2-	II	Tremelimumab Durvalumab Fulvestun	—	—	—	—
NCT03518606	All Types	I/II	Tremelimumab Durvalmab Metronomic Oral Vinorelbine	—	—	—	—
NCT03606967	TNBC	II	Tremelimumab Durvalumab Nab-Paclitaxel Personalized Synthetic Long Peptide Vaccine	—	—	—	—
NCT02453620	Metastatic or Unresectable or HER2-	I	Ipilimumab Nivolumab Entinostat	—	—	—	—
NCT02419495	TNBC	I	Ipilimumab Nivolumab Selinexor	—	—	—	—
NCT04185311	TNBC or ER+/HER2-	I	Ipilimumab Nivolumab Talimogene laherparepvec	—	—	—	—
NCT02833233	Early Stage	Not Applicable	Ipilimumab Nivolumab Cryoablation	—	—	—	—
NCT03546686	TNBC	II	Ipilimumab Nivolumab Cryoablation	—	—	—	—
NCT03409198	HR+or Metastatic	II	Ipilimumab Nivolumab Pegylated Liposomal Doxorubicin Cyclophosphamide	—	—	—	—
NCT03326258	TNBC	I/II	Ipilimumab Nivolumab CDX-011	—	—	—	—
NCT03058289	All Types	I/II	Ipilimumab Pembrolizumab INT230-6	—	—	—	—
NCT03755739	All Types	II/III	Ipilimumab Pembrolizumab Chemotherapy	—	—	—	—

These clinical trial data were obtained from https://clinicaltrials.gov.

### 5.2 Multiple combination therapy of anti-CTLA-4 antibodies and other drugs

There is a plethora of research on multiple combination therapies with anti-CTLA-4 antibodies + anti-PD-1/PD-L1 antibodies + other agents, and researchers have combined anti-CTLA-4 antibodies with multiple other agents for breast cancer treatment without the use of anti-PD-1/PD-L1 antibodies. BEmpeg, an IL-2 receptor agonist, stimulates the IL-2 pathway to elicit an antitumor response (85). Radiation therapy enhances the antitumor immune response to IL-2 therapy (86). Pieper et al. applied a combination of anti-CTLA-4 antibody, BEmpeg, and radiation therapy to a 4T1 breast cancer mouse model. The results demonstrated effective control of tumor growth and a significant improvement in mouse survival rates, indicating a potential novel immunotherapeutic approach (87).

To enhance the efficacy of ICIs, many scholars have investigated the combination of ICIs with nanoparticles and photothermal therapy. Li et al. designed a single-walled carbon nanotube (SWNT) modified by a novel immunoadjuvant glycated chitosan (GC). Mice with metastatic breast cancer were injected with anti-CTLA-4 antibody and SWNT-GC, and photothermal therapy was simultaneously applied. The results showed that the combination of these three treatments induced an antitumor immune response, reduced the probability of distant tumor metastasis, and improved the survival rate of breast cancer model mice (88).

McKernan also incorporated annexin A5 (ANXA5)-functionalized single-walled carbon nanotubes (SWCNTs) and photothermal therapy into the treatment of primary breast cancer in mice receiving anti-CTLA-4 antibody therapy. The survival time of the mice significantly increased, and their immune activity was greatly enhanced (89). Similarly, Yasothamani et al. designed a combination of hyaluronic acid (HA)-polyaniline (PANi)-imidazoquinoline (R837) nanoparticles, named HA-PANI/R837. Under photothermal stimulation, HA-PANI/R837 nanoparticles targeted tumor cells, facilitated the binding of anti-CTLA-4 antibody to R837, and then activated immunogenic cell death. The results showed a significant reduction in the number of tumor cells in the mice, indicating promising applications for the combination of ICIs, nanoparticles, and photothermal therapy (90). Additionally, Chen et al. evaluated the growth of 4T1 mouse breast tumors after combined treatment with iron oxide nanoparticle-mediated photothermal therapy and anti-CTLA-4 antibody. This combination therapy first eliminated Tregs associated with immune escape, subsequently inhibiting tumor expansion and promoting immune-based checkpoint blockade therapy (91).

## 6 Adverse events

In recent years, the application of ICIs in diseases has become more profound and extensive, making immunotherapy a breakthrough in cancer treatment. However, along with the beneficial therapeutic outcomes, irAEs have also emerged. A meta-analysis revealed irAEs caused by CTLA-4 and PD-1/PD-L1 inhibitors. Among them, the most common irAEs involve respiratory, thoracic, and gastrointestinal disorders. Patients below the age of 65 mainly experience reproductive system and breast diseases, while those above 65 predominantly exhibit cardiac diseases. Male patients are more prone to respiratory and thoracic disorders, whereas reproductive system and breast diseases prevail among female patients. The results indicate that sex or age contributes to the occurrence of different irAEs, requiring a heightened awareness of respiratory and genitourinary toxicities for male patients, while female patients need increased prevention of reproductive system toxicities (92). Additionally, multiple studies have demonstrated that ICIs can induce various dermatological adverse events (DAEs), such as autoimmune bullous dermatosis, acute generalized exanthematous pustulosis, and psoriasiform rash (93). According to several clinical trials of CTLA-4 inhibitors, breast cancer patients experience a variety of irAEs during treatment. The most common adverse events include anemia, hypothyroidism, diarrhea, fatigue, pain in the extremities, and dyspnea. Causes of death during treatment included the development of myasthenia gravis and urinary sepsis. During treatment with CTLA-4 inhibitors, patients should be constantly monitored for any signs or symptoms of irAEs. Physicians need to ensure that patients do not experience grade 3 or higher trAEs before continuing with immunotherapy. In cases of severe trAEs, immunotherapy should be discontinued, and high-dose corticosteroid therapy should be administered. The study of irAEs should be incorporated into systematic research to enhance our understanding of their mechanisms, thereby facilitating the development of appropriate tumor immunotherapy protocols and intervention strategies.

## 7 Future directions

The ongoing research and application of immunotherapy in breast cancer have substantially progressed. CTLA-4, a pivotal immune regulatory molecule, has already shown potential in breast cancer immunotherapy. There are promising prospects for CTLA-4-based breast cancer immunotherapy. Firstly, clinical trials need to further investigate the combination of CTLA-4 inhibitors with other immunotherapy drugs. These combination treatments have the potential to exert synergistic effects, trigger strong immune responses and enhance therapeutic outcomes for patients. Subsequently, the focal point will shift toward personalized treatment strategies as a pivotal avenue for future development. By carefully studying patient immune traits, genetic deviations, and the TME, scientists can precisely prognosticate patients’ responsiveness to CTLA-4 immune therapy. This method helps tailor the best treatment strategy for each patient during the regimen development phase, thereby optimizing efficacy while mitigating unwanted side effects. Furthermore, a more profound comprehension of the mechanisms governing CTLA-4 immunotherapy will facilitate the development of novel therapeutic paradigms. Researchers may develop drugs that target other key molecules in the CTLA-4 signaling pathway, thereby further improving efficacy. Simultaneously, the emergence of gene editing technologies and cell engineering paradigms has the potential to make immune cells more specific and viable, consequently expanding the sustained impact of immunotherapy. In conclusion, future directions for CTLA-4-based breast cancer immunotherapy include combination therapy and individualized treatment, and the complex mechanisms governing CTLA-4 immunotherapy will continue to be explored. These new directions will advance the development of breast cancer immunotherapy and provide more effective and targeted treatment options for patients.

## 8 Conclusion

Given the high incidence of breast cancer and the challenges in treating subtypes such as TNBC and HER2+ breast cancer, ICIs have gained considerable momentum in the treatment of breast cancer. The immunotherapeutic efficacy of CTLA-4 inhibitors in breast cancer has been validated in numerous clinical studies. However, the current use of CTLA-4 inhibitors as monotherapy for breast cancer still faces limitations in terms of response rates and common occurrences of drug resistance. Researchers have explored combining CTLA-4 inhibitors with other ICIs or drugs to enhance immune responses in breast cancer. While immunotherapy has substantial benefits, the occurrence of irAEs cannot be ignored. To improve the effectiveness of immunotherapy and mitigate its associated side effects, future efforts should focus on the development of safer and more effective combination strategies.

## Author contributions

HZ: Writing – original draft. JM: Writing – review & editing. QX: Writing – review & editing. WC: Writing – review & editing. CS: Writing – review & editing. NZ: Writing – review & editing. CY: Writing – review & editing.
